# Explore the Predictive Value of Peripheral Blood Cell Parameters in Refractory *Mycoplasma pneumoniae* Pneumonia in Children Over 6 Years Old

**DOI:** 10.3389/fped.2021.659677

**Published:** 2021-11-12

**Authors:** Yaoyao Ling, Jing Ning, Yongsheng Xu

**Affiliations:** Department of Respiratory, Tianjin Children's Hospital, Tianjin, China

**Keywords:** refractory *Mycoplasma pneumoniae* pneumonia, neutrophil/lymphocyte, mean platelet volume/lymphocyte, predictive value, children

## Abstract

**Background:** To determine the predictive value of peripheral blood cell parameters for refractory *Mycoplasma pneumoniae* pneumonia (RMPP) in children over 6 years old.

**Methods:** A retrospective study was conducted in children with RMPP admitted to the respiratory department of Tianjin Children's Hospital from September 2017 to September 2019, and non-refractory *Mycoplasma pneumoniae* pneumonia (NRMPP) was selected by the propensity score method and matched according to the ratio of 1:1.5. We analyzed the differences in clinical characteristics, peripheral blood cell parameters, imaging findings, and treatments between the two groups, and further determined the predictive value of peripheral blood cell parameters on RMPP.

**Results:** There were 76 patients in the RMPP group and 114 patients in the NRMPP group. We found that the RMPP group has a longer clinical course and a higher incidence of intrapulmonary and extrapulmonary complications (*p* < 0.01). Moreover, the proportion of children in the RMPP group who received immunotherapy (such as glucocorticoid, gamma immunoglobulin) and fiberoptic bronchoscopy intervention was higher than that in the NRMPP group (*p* < 0.01). Meanwhile, the level of neutrophil, neutrophil/lymphocyte ratio (NLR), platelet count/lymphocyte ratio (PLR), mean platelet volume/lymphocyte ratio (MPVLR), C-reactive protein (CRP), lactic dehydrogenase (LDH), and interleukin (IL)-6 in the RMPP group was significantly higher (*p* < 0.01) than those in the NRMPP group. The incidence of pulmonary consolidation, atelectasis, and pleural effusion was also higher in the RMPP group (*p* < 0.05). ROC curve and binary logistic regression analysis showed that NLR > 3.92 (OR = 3.243; 95% CI = 1.485–7.081; *p* = 0.003), MPVLR > 5.29 (OR = 2.700; 95% CI = 1.258–5.795; *p* = 0.011), and pleural effusion (OR = 3.023; 95% CI = 1.424–6.420; *p* = 0.004) were significant factors in predicting RMPP. Our study showed that NLR had higher accuracy in predicting RMPP than CRP.

**Conclusions:** The parameters of peripheral blood cells might be a predictor of RMPP. NLR > 3.92, MPVLR > 5.29, and pleural effusion might have important predictive value for RMPP in children over 6 years old.

## Introduction

*Mycoplasma pneumoniae* (MP) is a common pathogen of pediatric community-acquired pneumonia (CAP) (1). *Mycoplasma pneumoniae* pneumonia (MPP) is usually considered as a mild/self-limiting disease, which has a good response to macrolides, but sometimes it may develop into a refractory case. Refractory *Mycoplasma pneumoniae* pneumonia (RMPP) is mainly referred to as MPP characterized by persistent fever and progressive exacerbations of clinical symptoms, signs, and related imaging manifestations after standard treatment with macrolide drugs for more than 1 week (2). RMPP is more likely to cause severe pulmonary and extrapulmonary complications, including atelectasis, necrotizing pneumonia, encephalitis, arthritis, pericarditis, hemolytic anemia, and disseminated intravascular coagulation, and even endangers life (3–8).

Peripheral blood cell parameters, such as neutrophil/lymphocyte ratio (NLR), platelet count/lymphocyte ratio (PLR), and mean platelet volume/ lymphocyte ratio (MPVLR) are markers of systemic inflammation, and their changes are related to inflammatory stress response (9). In recent years, peripheral blood cell parameters have attracted more and more attention. A study showed that NLR contributes to risk stratification and prognosis in patients with acute coronary syndromes (10). Lattanzi et al. found that NLR was associated with 30-day mortality and morbidity after intracerebral hemorrhage, which could improve outcome prediction of acute intracerebral hemorrhage (11). They also played an important role in the prognosis of acute ischemic stroke patients undergoing revascularization treatment (12, 13), and had a certain predictive value in the occurrence, efficacy, and prognosis of tumors (14–16). The above evidence shows the significance and broad potential of serum biomarkers that are easy to obtain and inexpensive in daily clinical practice. Therefore, we aim to explore the predictive value of peripheral blood cell parameters in children with RMPP. Besides, due to the specificity of blood images of pre-school children, this study mainly explored the predictive value of peripheral blood cell parameters for children over 6 years old.

## Methods

### Study Population

A retrospective case–control study was conducted to select RMPP children admitted to the Respiratory Department of Tianjin Children's Hospital from September 2017 to September 2019, and non-refractory *Mycoplasma pneumoniae* pneumonia (NRMPP) was selected by the propensity score method and matched according to a ratio of 1:1.5.

### Diagnostic Criteria

All patients had signs and symptoms indicative of pneumonia (such as fever and cough) on admission, and pneumonic infiltrations in the chest radiograph. MP acute infection was based on the titer of MP-IgM antibody in single serum persisting at 1:160, and whether MP-DNA and/or MP-RNA was positive in nasopharyngeal secretions, or the titration of MP-antibody had a significant conversion in a double serum interval of 2 weeks (17). RMPP is mainly referred to as MPP characterized by progressive exacerbations of clinical symptoms, persistent fever, and aggravated lung imaging after standard treatment with macrolide drugs for 7 days or longer (2).

### Inclusion Criteria

(1) Children who meet the diagnostic criteria; and (2) are more than 6 years old but <15 years old.

### Exclusion Criteria

(1) Individuals with basic diseases such as asthma, chronic cardiopulmonary disease, rheumatism, and immune deficiency; (2) those with other respiratory pathogen infections and tuberculosis confirmed using the following tests: blood cultures, nasopharyngeal aspirate cultures, nasopharyngeal aspirate for virus reverse transcriptase real-time multiplex PCR, and serology of common respiratory pathogens; (3) those who had used glucocorticoids and antibiotics before the blood routine test; and (4) those whose clinical data were incomplete.

### Data Collection

Hospitalization demographics, clinical information, peripheral blood cell parameters, lactic dehydrogenase (LDH), interleukin (IL)-6, radiological findings, and treatments of all patients were collected in the study.

After admission, all patients had their body temperature (axillary temperature) monitored daily and their respiratory symptoms were evaluated, such as cough, wheezing, dyspnea, and chest pain. According to the eighth edition of Zhu Futang's Practical Pediatrics, a body temperature (axillary temperature) of above 37.5°C is defined as fever. Dyspnea refers to the exertion of breathing movement, which may result in mouth opening breathing, nasal flapping, inspiratory depression of chest wall, etc. We also evaluated extrapulmonary complications. Liver function damage is defined as an increase in alanine aminotransferase (ALT) of more than double the usual level. The diagnosis of thromboembolism is based on the related clinical symptoms of MPP children and imaging examination (such as ultrasound, CT, MRI, MRA, MRV, CTA, etc.) promoting thrombosis. Toxic encephalopathy refers to the symptoms of the nervous system, normal cerebrospinal fluid, negative etiological examination, and abnormal electroencephalogram; treatment of primary disease symptoms results in a gradual recovery. Skin and mucous membrane damage refers to systemic or local erythema multiforme, maculopapular and urticaria, and even Steven–Johnson syndrome.

We used the blood routine results of the first blood collection of the patients and calculated the related inflammatory indexes: NLR, PLR, and MPVLR. In addition, blood culture, sputum culture, sputum virus RT-PCR, blood biochemistry, LDH, IL-6, and blood pathogen examinations were completed within 24 h after admission.

Chest radiography was performed before admission or during hospitalization. Patients had a CT scan if he or she had one of the following situations: (1) the clinical manifestations were inconsistent with the chest radiograph; (2) airway and lung malformations were suspected; (3) there were serious complications associated with pneumonia; (4) patients failed to respond to treatment and other diseases needed to be excluded such as interstitial lung disease, pulmonary tuberculosis, and so on (18, 19).

In terms of treatment, the indications of glucocorticoids used in this study were as follows: (1) patients with obvious wheezes and increasing respiratory secretions; (2) severe pneumonia with obvious poisoning symptoms; (3) a lot of pleural effusion exudation for a short time; and (4) persistent high fever of pneumonia with strong inflammatory reaction (20). When combined with central nervous system disease, immune hemolytic anemia, immune thrombocytopenic purpura, and other autoimmune diseases, gamma immunoglobulin could be considered (20). Patients with atelectasis or inflammatory consolidation of one or more lung segments and no obvious improvement in chest imaging after routine intravenous anti-infection therapy could be considered for fiberoptic bronchoscopy therapy (20).

### Ethics

This study was approved by the medical ethics committee of Tianjin Children's Hospital, and informed consent was signed by the guardians of children before the collection of clinical case data.

### Data Analysis

SPSS 22.0 was used for statistical analysis. The normal distribution data were represented by mean ± SD ($\bar{x}$ ± *s*). The LSD *t*-test was used for comparison between the groups. Skewed distribution data were expressed as median (P25, P75), whose comparisons were made by the Mann–Whitney *U*-test. Chi-squared tests or Fisher's exact test were used to compare categorical data. Receiver operating characteristic (ROC) curves were calculated to determine the sensitivity and specificity of candidate peripheral blood cell parameters related to RMPP. Then, logistic regression analysis was carried out to further determine the independent variables related to RMPP. The difference was considered statistically significant at *p* < 0.05.

## Results

### Clinical Characteristics of Patients

A total of 190 patients were enrolled in this study, including 76 in the RMPP group and 114 in the NRMPP group. The average age was 7.93 ± 1.91 years old, and the ratio of boys to girls was about 1.26. There was no difference in age and gender distribution between these two groups. All patients had cough and fever. Besides, intrapulmonary and extrapulmonary complications were more common in the RMPP group, especially toxic encephalopathy, mucous plugging, and dyspnea (all *p* < 0.05) (Table 1).

**Table 1:** Clinical characteristic of RMPP and NRMPP patients.

**Clinical information**	**RMPP (*n* = 76)**	**NRMPP (*n* = 114)**	***P*-value**
Age, years	8.12 ± 2.06	7.81 ± 1.80	0.272
Male, *n* (%)	41 (53.95)	65 (57.02)	0.676
**Clinical presentation** ***n*** **(%)**			
Fever	76 (100.00)	114 (100.00)	1.000
Cough	76 (100.00)	114 (100.00)	1.000
Chest pain	4 (5.26)	1 (0.88)	0.084
Skin and mucous membrane	6 (7.89)	7 (6.14)	0.771
Thromboembolism	1 (1.32)	0 (0.00)	0.400
Wheezing	3 (3.95)	2 (1.75)	0.391
Dyspnea	10 (13.16)	0 (0.00)	0.000
Liver function damage	10 (13.16)	6 (5.26)	0.065
Toxic encephalopathy	4 (5.26)	0 (0.00)	0.024
Mucous plugging	28 (36.84)	0 (0.00)	0.000
Length of fever, days	11.5 (10.0-14.0)	9.0 (7.0-11.0)	0.000
Length of stay, days	10.0 (8.0-13.0)	6.0 (5.0-7.0)	0.000
**Management** ***n*** **(%)**			
Using azithromycin	76 (100.00)	114 (100.00)	1.000
Using glucocorticoids	66 (86.84)	29 (25.44)	0.000
Using gamma immunoglobulin	15 (19.74)	0 (0.00)	0.000
Using fiberoptic bronchoscope	63 (82.89)	35 (30.70)	0.000

### Clinical Course and Treatment of Patients

Regarding the clinical course, the median duration of fever in the RMPP and NRMPP groups was 11.5 (10.0–14.0) and 9.0 (7.0–11.0) days, respectively (*p* < 0.01). The median length of hospital stay was 10.0 (8.0–13.0) days in the RMPP group, and 6.0 (5.0–7.0) days in the NRMPP group (*p* < 0.01). Therefore, RMPP patients may be treated with more methods, such as glucocorticoids, immunoglobulin, and fiberoptic bronchoscopy (*p* < 0.01). All patients were treated with macrolide drugs (Table 1).

### Laboratory Parameters and Imaging Features of Patients

The average level of neutrophil count (N), MPV, NLR, PLR, and MPVLR in the RMPP group was 6.17 × 10^9^/L, 10.23 fl, 4.65, 219.37, and 8.50, respectively, which were higher than those in the NRMPP group (all *p* < 0.05). Lymphocyte count (L) was lower in RMPP, while no difference was found in total peripheral leukocyte count and platelet count. Besides, we compared the levels of C-reactive protein (CRP), LDH, and IL-6 between the two groups. The results showed that the levels of CRP, LDH, and IL-6 in the RMPP group were 43.55 mg/L, 493.26 IU/L, and 45.11 pg/ml, respectively, which were significantly higher than those in the NRMPP group (Table 2). In the study, all patients underwent chest X-ray examinations before or at admission. The percentage of CT scans in the RMPP group and the NRMPP group was 93.42 and 19.30%, respectively. Besides, the radiological abnormalities in the RMPP group were more serious. The incidence of pulmonary consolidation, atelectasis, and pleural effusion was higher in the RMPP group (all *p* < 0.05) (Table 3).

**Table 2:** Laboratory parameters of RMPP and NRMPP patients.

**Laboratory parameters**	**RMPP (*n* = 76)**	**NRMPP (*n* = 114)**	***P*-value**
WBC, × 10^∧^9/L	8.31 ± 3.92	7.80 ± 2.74	0.291
N, × 10^∧^9/L	6.17 ± 3.30	5.11 ± 2.22	0.000
L, × 10^∧^9/L	1.53 ± 0.75	1.97 ± 0.77	0.000
PLT, × 10^∧^9/L	280.87 ± 75.11	302.11 ± 96.75	0.108
MPV, fl	10.23 ± 0.93	9.96 ± 0.89	0.044
NLR	4.65 ± 2.59	2.94 ± 2.00	0.000
PLR	219.37 ± 112.45	167.98 ± 65.95	0.000
MPVLR	8.50 ± 5.30	5.95 ± 2.84	0.000
CRP (mg/l)	43.55 ± 42.08	21.66 ± 24.17	0.000
LDH (IU/L)	493.26 ± 212.85	369.35 ± 148.63	0.000
IL-6 (pg/ml)	45.11 ± 44.93	21.11 ± 31.29	0.000

*WBC, white blood cell; N, neutrophil count; L, lymphocyte count; PLT, platelet count; MPV, mean platelet volume; NLR, neutrophil/lymphocyte; PLR, platelet count/lymphocyte; MPVLR, mean platelet volume/lymphocyte; CRP, C-reactive protein; LDH, Lactic dehydrogenase; IL-6, Interleukin-6*.

**Table 3:** Radiological features of RMPP and GMPP patients.

**Radiological features**	**RMPP (*n* = 76)**	**NRMPP (*n* = 114)**	***P*-value**
Pulmonary consolidation	68 (89.47%)	86 (75.44%)	0.016
Lobar atelectasis	21 (27.63%)	12 (10.52%)	0.002
Pleural thickening	45 (59.21%)	58 (50.88%)	0.259
Pleural effusion	38 (50%)	20 (17.54%)	0.000

### Predictive Value of Peripheral Blood Cell Parameters in Patients With RMPP

**Figure 1:**
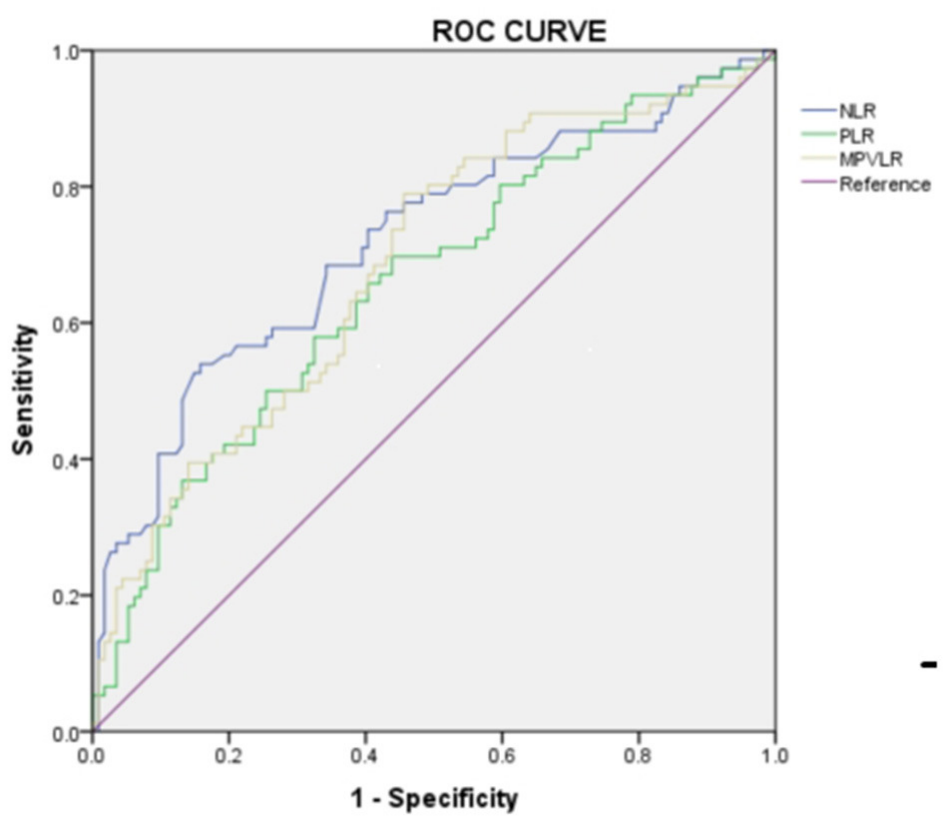
The ROC curve of predictive value of peripheral blood cell parameters in patients with RMPP.

**Figure 2:**
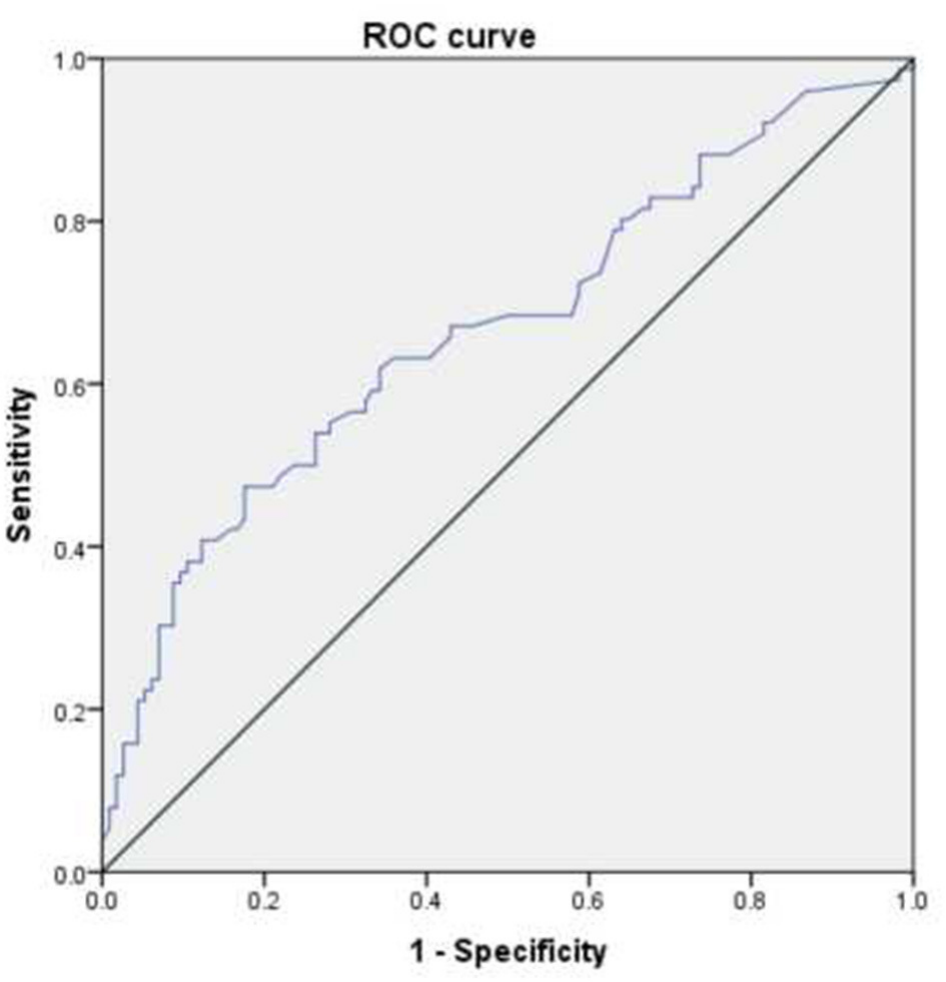
The ROC curve of predictive value of CRP in patients with RMPP.

To explore the predictive value of peripheral blood cell parameters in RMPP, a ROC curve (Figure 1) was calculated and the critical value with maximum sensitivity and specificity was determined. ROC curve analysis showed that NLR, PLR, and MPVLR were helpful to predict the incidence of RMPP. When the cutoff values of NLR, PLR, and MPVLR were 3.92, 159.56, and 5.29, the sensitivity and specificity were 0.539 and 0.842, 0.697 and 0.561, and 0.789 and 0.544, respectively. The areas under the curve of NLR, PLR, and MPVLR were 0.718, 0.658, and 0.685 (Table 4). In addition, we also conducted ROC analysis for CRP. The cutoff value, sensitivity, specificity, and the areas under the curve of CRP were 33.55 mg/L, 0.474, 0.825, and 0.667, respectively (Figure 2).

**Table 4:** Predictive value of peripheral blood cell parameters in patients with RMPP.

**Independent factors**	**Cut-off value**	**Sensitivity**	**Specificity**	**AUC**	***P*-value**	**95% CI**	
						**Lower**	**Upper**
NLR	3.92	0.539	0.842	0.718	0.000	0.642	0.795
PLR	159.56	0.697	0.561	0.658	0.000	0.579	0.738
MPVLR	5.29	0.789	0.544	0.685	0.000	0.607	0.762

### Multiple Logistic Regression Analysis for the Related Factors Predicting the Risk of RMPP

To further evaluate the predictors associated with RMPP, multiple logistic regression was performed. The results showed that NLR > 3.92 (OR: 3.243, *p* = 0.003), MPVLR > 5.29 (OR: 2.700, *p* = 0.011), and pleural effusion (OR: 3.023, *p* = 0.004) were significant predictors of the incidence of RMPP (Table 5).

**Table 5:** Multiple logistic regression analysis for the related factors predicting the RMPP.

**Variable**	**OR**	**95%CI**	***P*-value**
NLR	3.243	1.485-7.081	0.003
MPVLR	2.700	1.258-5.795	0.011
Pleural effusion	3.023	1.424-6.420	0.004

## Discussion

Although MP infection is a benign disease in most cases, the prevalence of RMPP has rapidly increased in recent years (21). It is significant for pediatricians to recognize RMPP earlier and treat it promptly. A blood routine examination is easy and low-priced. To the best of our knowledge, our study is the first to focus on the predictive role of peripheral blood cell parameters in children with RMPP.

So far, the pathogenesis of RMPP has not been fully understood. It is generally known that RMPP may be related to an excessive inflammatory reaction, MP resistance, abnormal secretion of airway mucus, mixed infection, and community-acquired respiratory distress syndrome toxins (22, 23). The host cell immune response can affect the clinical characteristics of MP infection. The proportion of pulmonary consolidation, atelectasis, and pleural effusion in the RMPP group was significantly higher than that in the NRMPP group, which was similar to the results of Zhang et al. (24). Children with RMPP were more likely to have extrapulmonary complications, such as dyspnea, toxic encephalopathy, and mucous plugging, which were mainly related to the immune response after MP infection. In addition, the days of fever and hospitalization in the RMPP group were significantly longer than that of the NRMPP group, which may lead to a stronger immune-inflammatory reaction, so more complex treatments should be performed. The proportion of the RMPP group receiving glucocorticoids and fiberoptic bronchoscopy treatment was higher, and some children with RMPP (19.75%) also used gamma immunoglobulin treatment.

Neutrophils and lymphocytes are essential cellular components of human host defense. Neutrophils are the first cell line of defense against infection in the non-specific immune system. They induce and activate an inflammatory response, while lymphocytes play a role in adaptive immunity (25). In our study, the average levels of neutrophils and lymphocytes in the RMPP group were 6.17 × 10^9^/L and 1.53 × 10^9/^L, respectively. This was similar to other studies where the children with RMPP showed a higher proportion of neutrophils and a lower percentage of lymphocytes (26, 27). NLR refers to the ratio of neutrophils to lymphocytes in peripheral blood. Our study showed that the children with RMPP had a higher NLR value. Nowadays, many studies have shown the prediction of CRP in RMPP (28–30). In ROC curve analysis, the area under the curve of CRP was 0.667, which was lower than that of NLR (0.718). Therefore, NLR had a higher accuracy rate than CRP in predicting the incidence of RMPP. It has been reported that NLR could better predict the clinical outcomes of patients with systemic inflammation (31). Besides, NLR is a more easily quantified biomarker than other inflammatory factors, such as tumor necrosis factor and interleukin (32). This may indicate the role of excessive inflammatory response in RMPP. MP infection can stimulate the activation of T lymphocytes, promote the high expression of inflammatory factors and cytokines, and further aggravate the body's inflammatory response (33). However, an excessive inflammatory reaction may activate apoptosis of lymphocytes, resulting in the decrease of lymphoid count (25).

Platelets are considered to be inflammatory reactants in the acute phase, which can release inflammatory mediators, such as chemokines and cytokines, thus aggravating the inflammatory response (34). MPV is a marker of platelet function and activation (35), which may be more sensitive than platelets. We found that PLR and MPVLR in the RMPP group were significantly higher than those in the NRMPP group, which may further suggest that there is an excessive inflammatory reaction in RMPP.

To better predict the predictive value of peripheral blood cell parameters, we conducted ROC curves and logic regression analysis. The results showed that NLR > 3.92 (OR = 3.243; 95% CI = 1.485–7.081; *p* = 0.003), MPVLR > 5.29 (OR = 2.700; 95% CI = 1.258–5.795; *p* = 0.011), and pleural effusion (OR = 3.023; 95% CI = 1.424–6.420; *p* = 0.004) had a positive value in predicting the risk of disease. Besides, the areas under the curve of NLR and MPVLR were 0.718 and 0.685, respectively.

Many studies have explored some important predictive indicators of RMPP, including C-reactive protein, lactate dehydrogenase, and interleukin-6 (24, 36, 37). There has been no study until now showing that peripheral blood cell parameters could be significant factors in predicting the risk of RMPP, and that they have the advantages of low cost and simple operation.

### Limitations and Strengths

The advantage of our study was exploring the predictive effect of peripheral blood cell parameters on RMPP for the first time, which was easy to obtain. Besides, we excluded the influence of age on peripheral blood cells. However, our study was a retrospective study, and the subjects may have selective bias. In future work, we should further carry out long-term multi-center and larger sample prospective studies to further prove the predictive value of peripheral blood cell parameters on RMPP.

## Conclusion

Our study showed that excessive inflammatory response might play an important role in RMPP and the parameters of peripheral blood cells might be a predictor of RMPP. NLR > 3.92, MPVLR > 5.29, and pleural effusion might have important predictive value for RMPP in children over 6 years old.

## Data Availability Statement

The original contributions presented in the study are included in the article/supplementary material, further inquiries can be directed to the corresponding author/s.

## Ethics Statement

Written informed consent was obtained from the individual(s) for the publication of any potentially identifiable images or data included in this article.

## Author Contributions

YL and YX: conception and design. YL: extraction of data and drafting the article. JN and YX: revising the article for intellectual content. YL, JN, and YX: final approval of the completed article. All authors read and approved the final manuscript.

## Funding

This work was supported by Tianjin Science and Technology Committee (20JCZXJC00170).

## Conflict of Interest

The authors declare that the research was conducted in the absence of any commercial or financial relationships that could be construed as a potential conflict of interest.

## Publisher's Note

All claims expressed in this article are solely those of the authors and do not necessarily represent those of their affiliated organizations, or those of the publisher, the editors and the reviewers. Any product that may be evaluated in this article, or claim that may be made by its manufacturer, is not guaranteed or endorsed by the publisher.

## Abbreviations

MP: *Mycoplasma pneumoniae*
MPP: *Mycoplasma pneumoniae* pneumonia
RMPP: refractory *Mycoplasma pneumoniae* pneumonia
NRMPP: non-refractory *Mycoplasma pneumoniae* pneumonia
N: neutrophil count
L: lymphocyte count
MPV: mean platelet volume
NLR: neutrophil/lymphocyte ratio
PLR: platelet count/lymphocyte ratio
MPVLR: mean platelet volume/lymphocyte ratio
CRP: C-reactive protein
LDH: lactic dehydrogenase
IL-6: interleukin (IL)-6
PCR: polymerase chain reaction
ROC: receiver operating characteristic.
